# Full recovery of lung tissue after severe viral pneumonia H1N1: A case report with 10 years follow-up

**DOI:** 10.1097/MD.0000000000033052

**Published:** 2023-02-22

**Authors:** Ladislav Kočan, Jozef Firment, Ingrid Pirníková, Silvia Farkašová Iannaccone, Dušan Rybár, Juliána Gnoriková, Ján Korček, Hana Kočanová, Pavol Török, Simona Rapčanová, Janka Vašková

**Affiliations:** a Clinic of Anaesthesiology and Intensive Care Medicine, East Slovak Institute of Cardiovascular Disease, Košice, Slovak Republic; b Ist Clinic of Anestesiology and Intensive Medicine, Faculty of Medicine, Pavol Jozef Šafárik University in Košice and Louis Pasteur University Hospital, Košice, Slovak Republic; c Department of Forensic Medicine, Faculty of Medicine, Pavol Jozef Šafárik University in Košice, Košice, Slovak Republic; d Department of Anaesthesiology and Intensive Care Medicine, Štefan Kukura Hospital with Policlinic, Michalovce, Slovak Republic; e Clinic of Anaesthesiology and Intensive Care Medicine, Railway Hospital and Clinic Košice, Slovak Republic; f Europainclinics, Bardejov, Slovak Republic; g Department of Medical and Clinical Biochemistry, Faculty of Medicine, Pavol Jozef Šafárik University in Košice, Košice, Slovak Republic.

**Keywords:** covid-19, H1N1, lung fibrosis, pneumonia, recovery, viral infection

## Abstract

**Rationale:**

World healthcare frequently faced severe viral pneumonia cases in the last decades, due to pandemic situations such as H1N1, MERS-CoV, and SARS-COVID-19.

**Patient concerns:**

The impact of viral infection on lung structure, lung function, and overall mortality was significant. The quality of life and assumed life expectancy was decreased with the supposed development of lung fibrosis in involved survived patients.

**Diagnoses:**

We described the course and treatment of severe pneumonia H1N1 in a 30-year-old patient.

**Interventions:**

Patient was included in a study regarding the therapeutic efficacy of selenium ClinicalTrials.gov ID: NCT02026856 with 10 years follow-up with concurrently documented X-ray lung examinations and final histology of lung tissue after sudden death.

**Outcomes:**

All sequential examinations and histological findings show a healing trend with the final full recovery of lung tissue.

## 1. Introduction

Pneumonia causes high morbidity and mortality with high hospitalization rate worldwide. Viral pneumonias are characterized by epidemic and pandemic spread in certain time cycles. Examples are the Spanish flu (1918–1920) with an estimated 500 million infected, 50 to 100 million victims, the Asian flu (1957–1958) responsible for 1 to 1.5 million deaths, or the Hong Kong flu, which broke out in in 1969, with an estimated 750 million infected patients and up to 1 million victims.^[1]^ In the interpandemic period, viral pneumonias are considered less serious compared to bacterial pneumonias. However, mortality from viral lung infections has increased dramatically in the last decade with the emergence of new strains of viruses such as the pandemic A type of influenza A virus, swine flu (H1N1) 2009 virus, which is a combination of swine, avian and human influenza viruses, and as well as the emergence of a new strain of the Middle East respiratory syndrome Coronavirus in the Middle East.^[2]^ Since 2019, the human society has faced a new pandemic threat caused by the severe acure respiratory syndrome-related Coronavirus 2 (SARS-CoV-2) virus, in various evolving variants. The manifestation of the infection is the disease COVID-19 with the dominance of respiratory symptoms, including viral pneumonia.^[3]^

The treatment strategy of mild respiratory forms of COVID-19 to severe clinical conditions associated with respiratory failure with the development of the acute respiratory distress syndrome and associated complications has brought a spectrum of new therapeutic procedures. At the same time, new data on the health status of patients after overcoming more severe forms of COVID-19 point to a new problem, which is a kind of “hidden pandemic” of post-COVID consequences. The conclusions of observational clinical studies show that up to 90% of patients hospitalized with COVID-19 have symptoms of dyspnea, reduced diffusion capacity of the lungs, related to lung tissue damage, and subsequently a decrease in physical activity after infection. In more than 50% of patients symptoms disappear within 3 months, while it is assumed that these patients are going to have a complete regeneration of lung tissue within 9 months after the end of hospitalization. A significant part of the population has some pre-disease lung tissue damage already, and almost 10% of all patients show fibrotic lung damage even before the infection of COVID-19. Predisposed patients are exposed to a greater risk of developing post-COVID fibrotic remodeling of the lung parenchyma. The 2% to 6% incidence of post-COVID pulmonary fibrosis can be estimated after moderate respiratory involvement. The rate of lung remodeling increases with the severity of the disease course.^[4]^ However, not all clinically serious pneumonias caused by viruses lead immediately to fibrosis.

The case report shows a retrospective view of the regenerative processes of the patient’s lung parenchyma after an H1N1 infection with acute respiratory distress syndrome in the acute phase of the disease, his physical lung computed tomography (CT) and X-ray findings during the next 10 years of follow-up, and histological findings of lung tissue from the patient’s autopsy.

## 2. Case report

**Figure 1.**
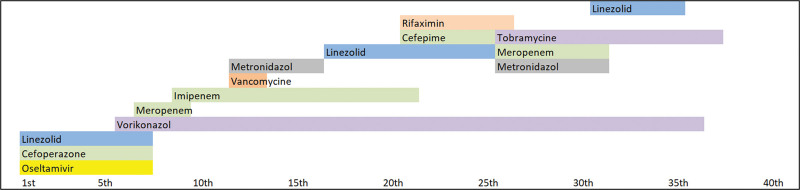
Time sequence of dynamic changes in antimicrobial treatment according to empirical experiences of the Intensive Care Unit, prospectively medication followed according to microbial culture and its sensitivity, and individual patient toleration to drugs.

A 30-year-old patient hospitalized in January 2011 at the Intensive Care Unit of University Hospital due to bilateral pneumonia presented with respiratory failure with an oxygenation index < 200. At admission, intubation and initiation of controlled ventilation was started. Pressure controlled ventilation with fraction of inspired oxygen of 0.8, positive end-expiratory pressure (PEEP) 9 cm H_2_O, pressure controlled ventilation 22 cm H_2_O and minute ventilation 12 l/minute was applied along with sedation, continuous administration of muscle relaxants, and prone positioning. Vasopressor support with a norepinephrine dose of up to 0.15 µg/kg/minute was necessary to stabilize circulation. Empirical antibiotic treatment and anticoagulation therapy was started (Fig. 1).

Viral infection of influenza A type H1N1 was diagnosed by the PCR method, thus a virostatic agent Oseltamivir was added. Restrictive fluid administration was maintained daily. Antifungal agent Voriconazol was later added for the positive galactomannan test. The patient’s condition deteriorated as he developed severe sepsis. *Pseudomonas aeruginosa* was confirmed from the bronchoalveolar lavage leading to the adjustment of antibiotic treatment (Fig. 1) and adding immunomodulation therapy with intravenous immunoglobulin and Polyoxidonium. On the 5th day the so called programmed multilevel ventilation mode performed by the Chirana AURA V (Slovakia) ventilator was applied for the first time with basic PEEP level 7 to 9 cm H_2_O, pressure control 12 to 10 cm H_2_O, frequency of 22 to 23/minute, upper PEEP level of 5 to 6 cm H_2_O was applied 4 to 5 times per minute, resulting in minute ventilation of 12 to 8 l/minute. Cortisol therapy started from the 5th day of hospitalization in a daily dose of 200 mg, later reduced to 100 mg, followed by reduction to 50 mg per day during the overall 15-day corticosteroid therapy. On 5^th^ day, selenium adjuvant therapy started too. Patient was enrolled in the study Se-AOX approved by the ethical committee of Pavol Jozef Šafárik University in Košice under number 109/2011; ClinicalTrials.gov Identifier: NCT02026856. Selenium in the form of sodium selenite pentahydrate at 750 mg/day for 6 days as a continual infusion was supplemented (1000 mg of sodium selenite pentahydrate = 333 μg of selenium) (Selenase, Vivax). During the severe sepsis, renal insufficiency developed with oliguria and an increase in blood levels of the urea and creatinine which necessitated the initiation of continuous veno-venous hemodialysis. Diarrhea developed 11 days from admission.

**Figure 2.**
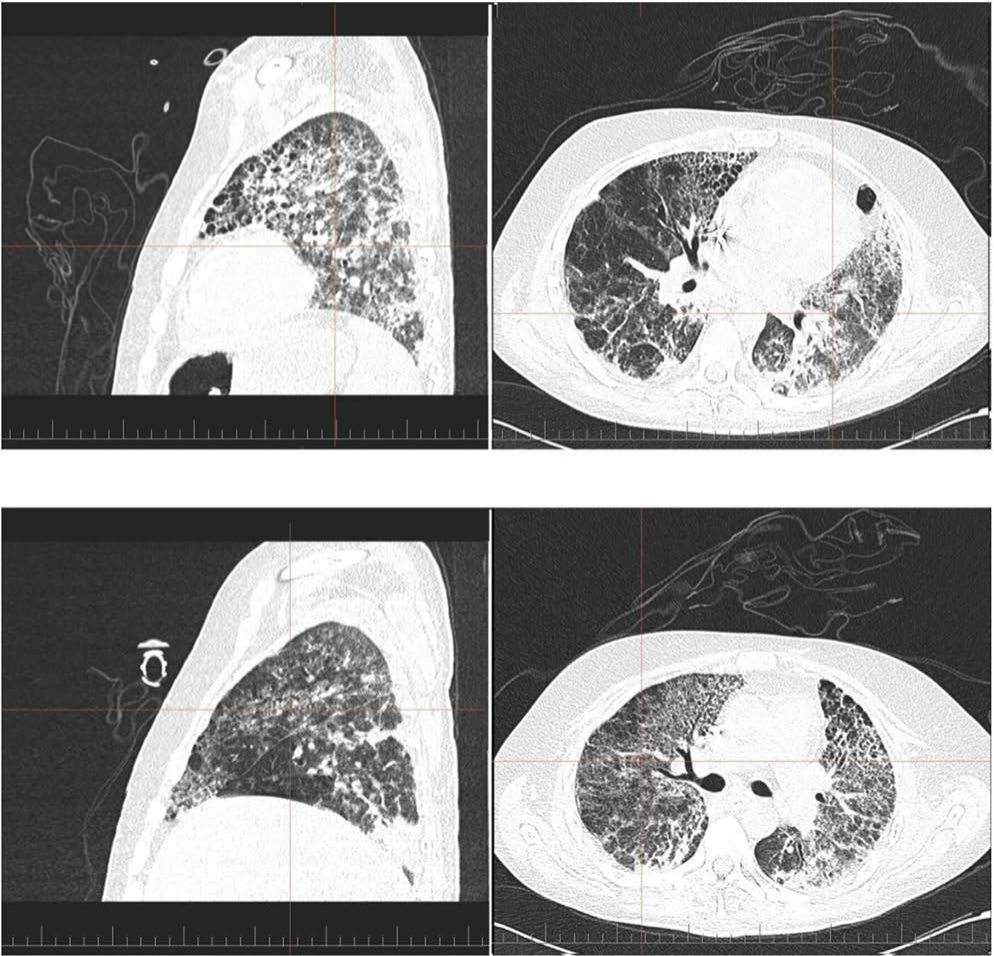
CT sagittal and a coronal view of lungs. In the upper row, the red cross marker is focused on the left lung. In lower row, the red cross marker is focused on the right lung. CT = computed tomography.

In the next course, the patient´s lung compliance and resistance started to improve. A gradual ventilation weaning was started by switching the 3-level ventilation to pressure control and later to pressure support ventilation mode with Pps 10 to 6 cm H_2_O, PEEP 7 to 5 cm H_2_O and minute ventilation 10-7 l/minute. On the 14^th^ day a CT scan of the lungs showed bilaterally present diffuse opacities of the milk glass type with thickening of the interlobular septation (Fig. 2). Peribronchovascular irregular consolidations were present in the upper and posterobasal lung segments of the lower lung lobes with a negative bronchogram. In superposition with the described changes, there was a suspicion of micro- and macrocystic remodeling of the lung parenchyma (so called honeycombing) on the periphery of the left lingular lung segments and the right medial segment of the middle lung lobe. The pleural spaces were free from fluid effusions. On the 15^th^ day of hospitalization, percutaneous dilatation tracheostomy was performed. 17 days after admission, clipping was attempted for gastrointestinal bleeding from a duodenal ulcer- unsuccessfully, therefore, and a laparotomy with successful duodenotomy was performed in the D2 to 3 region. In the postoperative period, paralytic ileus developed and was treated conservatively. Subsequently, enteral nutrition was started once again. In the next course of hospitalization, repeated attempts to wean the patient from controlled ventilation were made. Concurrently, withdrawal symptoms appeared after long-term application of opioids, therefore, and neuroleptics were started. The patient gradually improved and was able to breathe spontaneously for several hours a day through a T-tube with intermittent pressure support ventilation. Continuous renal replacement therapy was switched to intermittent hemodialysis. After repeated negative cultures from biological samples, antibiotic treatment was terminated. At that time, the patient was on an oral full-fledged diet and adhered to a complex rehabilitation process. In the following days, the patient developed a polyuric phase of acute kidney injury followed by a gradual recovery of renal functions. On the 47^th^ day, the patient was transferred to the intensive care unit of the regional hospital. The patient further improved and was discharged to outpatient care.

In 2012 and 2014 the patient was treated by the emergency medical service for difficult breathing leading to admission to the local hospital’s internal medicine department.

**Figure 3.**
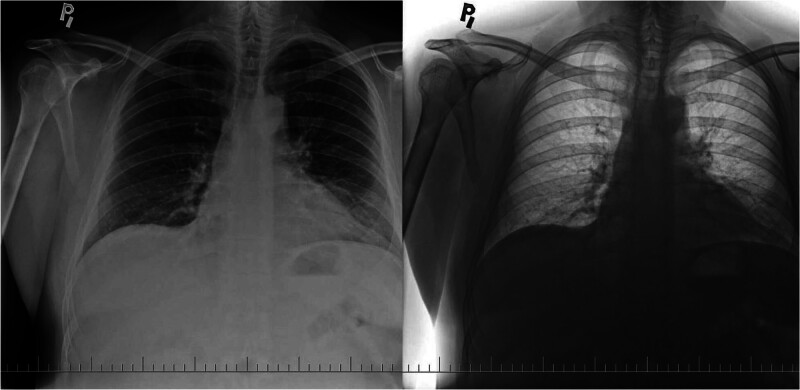
Chest X-ray in Posterior-Anterior projection of lungs. April 2018 local hospital’s surgical department.

**Figure 4.**
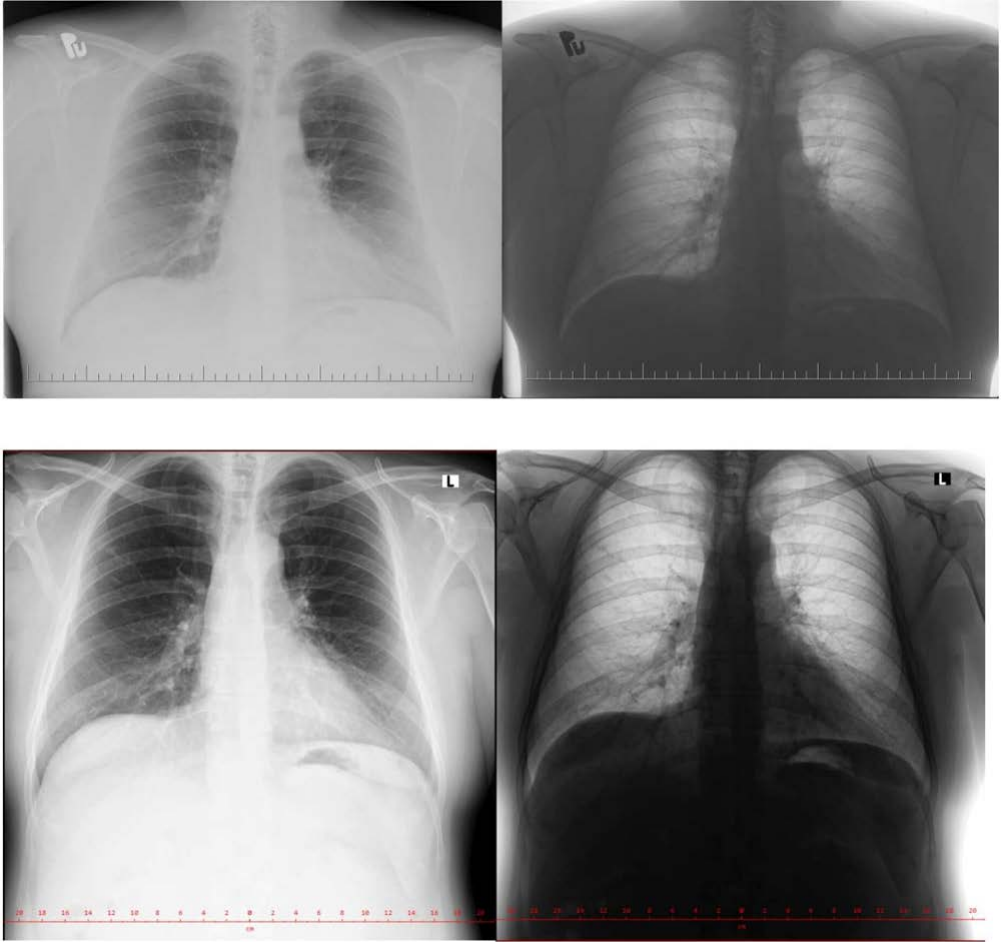
A: Chest X-ray in Posterior-Anterior projection of lungs. May 2019 local hospital’s surgical department. B: Chest X-ray in Posterior-Anterior projection of lungs. March 2021 local hospital’s surgical department.

In April 2018, the patient was hospitalized in the local hospital’s intensive care unit due to acute pancreatitis as a result of excessive alcohol consumption, confirmed by laboratory and CT examination. The condition was complicated by the development of disorientation, agitation, and delirium, which necessitated the use of sedatives and antipsychotics. Despite the treatment, the condition did not improve, furthermore, and respiratory insufficiency developed. The patient was intubated and controlled ventilation was started and vasopressors were temporarily used. The following day, the symptoms improved, sedation was gradually discontinued, ventilation support was reduced and finally the patient was extubated. He was transferred to the surgical department. The control X-ray of the chest from this period describes findings without serious pathology, presenting indistinct irregularities of the course bronchovascular pattern in the lower lung fields (Fig. 3). The patient was discharged home after 7 days. Control X-ray examination in following 2-years period shows Figure 4.

In November 2021, the patient was transported by the emergency medical service to the local hospital’s emergency department after falling on the street. Coincidentally, a diagnosis of COVID-19 by antigen testing was made. The patient was examined by a traumatologist, a neurologist, and an internist. A small subarachnoid hemorrhage was found on the left temporal side on the head CT scan. Laboratory results showed a significant elevation of hepatic enzymes, leukopenia, thrombocytopenia, moderate elevation of C-reactive protein, D-dimers, and fibrinogen. Abdominal ultrasound revealed cirrhosis of the liver. The patient had repeated grand mal seizures and developed delirium. In the evening, a cardiac arrest suddenly occurred. Immediately, advanced cardiopulmonary resuscitation was started but was unsuccessful and the patient died. An autopsy was indicated.

**Figure 5.**
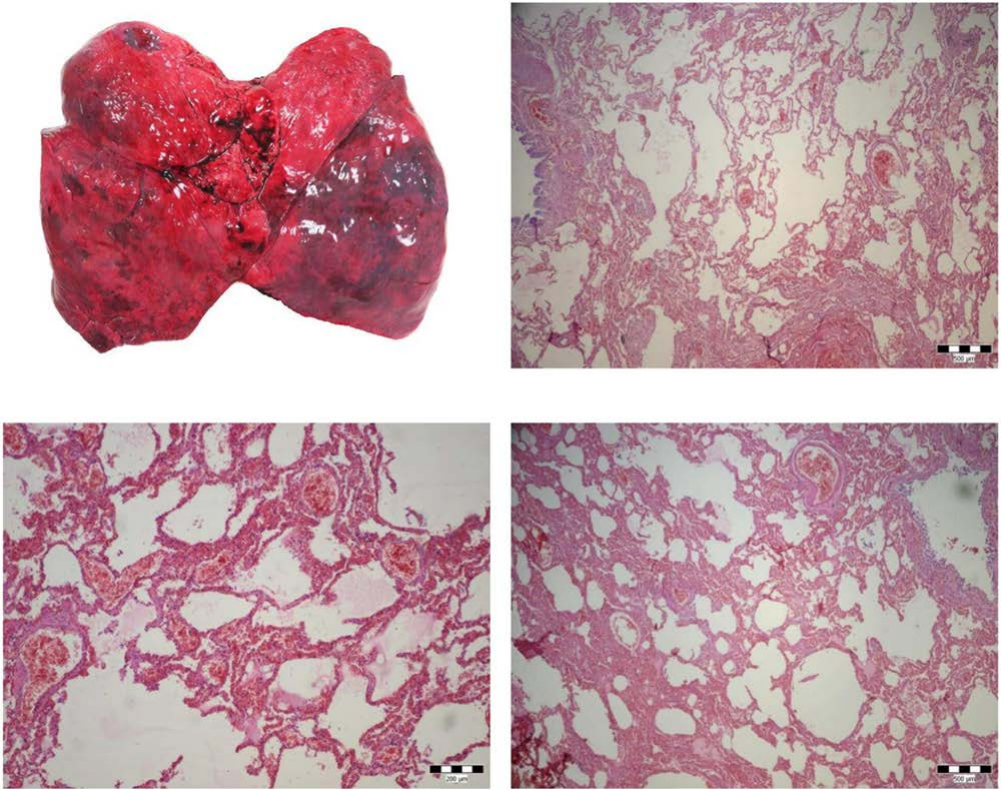
A: Lungs: the upper lobes with dilatation of air spaces; the lower lobes were dry, firm, airless and consolidated. B: Lungs: acute congestion, focal hemorrhage, focal emphysema, mild alveolar edema, scant interstitial lymphoplasmacytic infiltrate, some interstitial fibrosis (hematoxylin and eosin, x40). C: Lungs: acute congestion, hemorrhage, mild alveolar edema, focal emphysema (hematoxylin and eosin, x100). D: Lungs: acute congestion, focal hemorrhage, focal emphysema, mild alveolar edema, scant interstitial lymphoplasmacytic infiltrate, some interstitial fibrosis (hematoxylin and eosin, x40).

Autopsy was performed at the Medico-Legal and Pathological-Anatomical Department of Health Care Surveillance Authority in Košice within 24 hours after death. Death was attributed to severe cerebral edema following traumatic brain injury (subarachnoid hemorrhage, focal contusions of the brain). The right and left lungs weighted 535 g and 515 g respectively. The pleural surfaces were smooth with a mild accumulation of anthracotic pigment deposited along lymphatic routes in the pleura (anthracosis). The upper lobes demonstrated dilatation of air spaces; the lower lobes were dry, firm, airless and consolidated. The parenchyma of the lower lobes was pink-red to red-purple in color with a meaty appearance and showed no expression of fluid from the cut surfaces upon compression (Fig. 5). The bronchi were white-gray with no sign of fluid collection. Histological examination of hematoxylin-eosin stained sections taken from both lungs (1x right upper lobe, 1x right middle lobe, 1x right lower lobe, 1x left upper lobe, 2x left lower lobe) revealed acute congestion, hemorrhage, focal emphysema, mild alveolar edema, and scant interstitial lymphoplasmacytic infiltrate and some interstitial fibrosis. There were no signs of characteristic histologic features of viral pneumonia (e.g., influenza pneumonia, SARS) such as diffuse alveolar damage (hyaline membranes, type II. pneumocyte hyperplasia, fibrin thrombi in small pulmonary arteries) and prominent interstitial lymphoplasmacytic infiltration. Neutrophil-rich intra-alveolar exudate suggestive of bacterial pneumonia was not observed. Reparative responses (squamous metaplasia, dense fibrosis) were not present. In this case, histologic features were not suggestive of a previous viral lung infection.

## 3. Discussion

Pulmonary fibrosis is a chronic complication of severe viral pneumonia, often accompanied by varying degrees of respiratory insufficiency. However, CT findings of the lungs of these patients indicate that signs of pulmonary fibrosis may subside over time.^[4]^ The mentioned case shows the high regenerative capacity of the lungs after overcoming severe viral pneumonia, while the initial prognostic assumption was the fibrotic remodeling of the lung tissue. This topic is relevant due to the large percentage of patients who have overcome the COVID infection and the further prediction of their prognosis.

In general, lungs are very fragile against various noxae. Vulnerability stems from the very structure of the lung tissue, which is the low density of cells in relation to the lung volume. Lung function depends on the arrangement of cells forming alveolar septa.^[5]^ The entry gate of the SARS Cov-2 virus into the cells of the respiratory system is the membrane receptor for angiotensin-converting enzyme 2 or the membrane protein transmembrane serine protease 2. The virus is able to infect alveolar type I cells (AT1) and alveolar type II cells (AT2). It is believed that only a small population of AT2 cells express the angiotensin-converting enzyme 2 receptor on their surface.^[6]^ The most vulnerable cells are located on the surface of the alveoli.^[7]^ Damage to AT1 stimulates the rapid proliferation and differentiation of AT2 cells, which also regenerates the tissue barrier function.^[5]^ Alveolar regeneration after acute lung injury has been observed in animal studies demonstrating the key function of AT2 cells. AT2 cells, as basic structural components of the alveolar epithelium, are able to further proliferate and differentiate into AT1 cells. AT1 cells form the functional structure of the alveoli. However, evidence of lung tissue regeneration in humans is absent, probably due to the lack of lung samples obtained. The SARS-CoV-2 virus can directly infect AT2 pneumocytes and cause their massive apoptosis. Inflammatory mechanisms play an important role in this process. It is not known whether alveolar regeneration occurs in the same way in humans after lung injury induced by SARS-CoV-2 infection as in animal models. The time interval of regeneration is also questionable. In rodent animal models, lung regeneration and recovery of function takes several weeks.^[8]^

Studies dealing with the recovery of respiratory functions in humans point to processes that last several years, which implies a longer onset of differentiation of AT2 cells, in contrast to animal studies on rodents, in which this process lasts only a few weeks.^[8]^ In case of long-lasting and destructive damage to the lungs, the formation and accumulation of fibrous connective tissue (fibrosis) occurs in the places of damage. It is a reparative mechanism in which fibrous tissue is formed. Accumulation of fibrous tissue at the site of damage to the epithelium and endothelium with a significant reduction in lung function and increased morbidity is characteristic. Pulmonary fibrosis is a common outcome of most chronic inflammatory lung disorders and can affect lung function, ultimately leading to respiratory failure and death.^[9]^ The process of fibrous tissue formation also depends on the severity and duration of the injury, as long-lasting injuries tend to develop into fibrosis in contrast to small-scale injuries. Interactions between different cell types have been found to be very important for the onset of fibrosis.^[8]^ Mesenchymal cells and fibroblasts are considered to be key in this process. Other important processes are the activation of glycolysis in fibroblasts after lung injury and a cascade of enzymatic activations that increase cell proliferation, collagen synthesis, and production of secondary metabolites, thereby promoting fibrosis. Increased glutaminolysis and fatty acid oxidation play an important role in the activation of fibroblasts. At the same time, resident monocytes and macrophages have a regulatory role in tissue fibrosis processes. They help initiate, maintain, and repair tissue damage.^[8,10]^

The level of the inflammatory response is a decisive factor whether regeneration or fibrosis occurs. Both processes of damaged tissue replacement are often sequential and interconnected. In the case of limited damage, regeneration usually occurs preferentially to restore tissue integrity and function. If this process fails due to severe damage, fibrous tissue formation is initiated, and which can lead to chronic lung disease or collapse. This suggests that regeneration is a full-fledged process, while fibrous tissue formation is only good when it is moderate. In the case of viral infection, and inflammatory damage is prominent and fibrosis is the dominant process before regeneration.^[10,11]^ Factors such as the amount and types of cells that are damaged, the disruption of barrier function, the intensity and duration of the local immune response can be a predictor of whether the damaged lung regenerates ad integrum or the formation of fibrous tissue leads to chronic lung disease.^[5]^ Study Se-AOX, in which this patient was also included, pointed out, for example, to a significant improvement of respiratory functions quantified on the basis of Carrico index in early selenium adjuvant therapy.^[12]^

## 4. Conclusion

The case report of a patient affected by severe viral pneumonia points to an example of possible complete regeneration of lung tissue, despite severe lung damage. Pathological changes in cellular organization within reparations include the formation of fibrous connective tissue, which inevitably changes the critical structure of lung properties and leads to poorer lung function. However, clinical experience and several studies have shown that the respiratory system has an extensive capacity to respond to noxae by regenerating damaged cells or proliferating and differentiating progenitor cells or changing the function of already existing differentiated cells. Pathological studies show that in damaged lung tissue, AT2 cells are the most extensive proliferating population in severely damaged lungs. Alveolar regeneration can be initiated especially in patients with less severe viral pneumonia.

## Author contributions

**Conceptualization:** Ladislav Kočan, Jozef Firment, Pavol Török, Janka Vašková.

**Data curation:** Ladislav Kočan, Jozef Firment, Ingrid Pirniková, Silvia Farkašová Iannaccone, Dušan Rybár, Juliána Gnoriková, Ján Korček, Pavol Török.

**Formal analysis:** Ladislav Kočan, Ingrid Pirniková, Silvia Farkašová Iannaccone, Dušan Rybár, Juliána Gnoriková, Ján Korček, Hana Kočanová, Pavol Török, Simona Rapcanova, Janka Vašková.

**Investigation:** Ladislav Kočan, Jozef Firment, Janka Vašková.

**Methodology:** Ladislav Kočan, Juliána Gnoriková, Ján Korček.

**Supervision:** Jozef Firment, Silvia Farkašová Iannaccone.

**Validation:** Ladislav Kočan, Jozef Firment, Ingrid Pirniková, Simona Rapcanova.

**Writing – original draft:** Ladislav Kočan, Silvia Farkašová Iannaccone, Dušan Rybár, Hana Kočanová, Pavol Török, Janka Vašková.

**Writing – review & editing:** Ladislav Kočan, Jozef Firment, Silvia Farkašová Iannaccone, Hana Kočanová, Simona Rapcanova, Janka Vašková.
